# Integrated multi-omics analysis reveals dendritic cell centered regulatory networks and therapeutic targets in necrotizing enterocolitis

**DOI:** 10.3389/fimmu.2026.1900420

**Published:** 2026-08-07

**Authors:** Feng Chen, Faling Chen, Yeerfan Aierken, Li Lu, Qingqi Chong, Tingting Gao, Tao Liu, Zhiru Wang, Zhibao Lv, Ting Guo

**Affiliations:** Department of General Surgery, Shanghai Children’s Hospital, School of Medicine, Shanghai Jiao Tong University, Shanghai, China

**Keywords:** bioinformatics, dendritic cells, immune microenvironments, intestinal barrier, necrotizing enterocolitis

## Abstract

**Background:**

Necrotizing enterocolitis (NEC) is a severe inflammatory intestinal disease predominantly affecting premature infants and is characterized by immune dysregulation and epithelial barrier disruption. However, the cellular communication networks and key molecular regulators underlying NEC pathogenesis remain incompletely understood.

**Methods:**

Single-cell RNA sequencing (scRNA-seq) and transcriptomic datasets of NEC were obtained from the Gene Expression Omnibus (GEO) database and analyzed using R-based bioinformatic workflows to characterize cellular composition, intercellular communication, immune infiltration, pathway activity, and candidate disease-associated genes. The expression of alpha kinase 2 (ALPK2) was validated in NEC intestinal tissues by quantitative real-time PCR, and its potential functional relevance was further investigated using lipopolysaccharide (LPS)-induced intestinal organoids.

**Results:**

Dendritic cells (DCs) occupied a central position in the NEC intercellular communication network and were enriched in antigen presentation and immune regulatory pathways. Machine learning identified ALPK2, ERICH1, and TMEM123 as candidate NEC-associated genes. Immune infiltration analysis revealed a disturbed immune microenvironment in NEC, with ALPK2 and ERICH1 negatively correlated with DC abundance. Functional enrichment analyses suggested that ALPK2 was predominantly associated with immune and inflammatory pathways, whereas ERICH1 was linked to metabolic and proliferative programs. ALPK2 expression was significantly elevated in ileal tissues from NEC patients and experimental NEC mice. In an inflammation model of LPS stimulation using mouse intestinal organoids, TGX221 suppressed inflammatory cytokine induction and partially restored tight junction protein expression.

**Discussion:**

These findings highlight DCs as central mediators of NEC-associated immune communication and identify ALPK2 as a potential regulator of intestinal inflammation and epithelial barrier dysfunction, supporting ALPK2 as a candidate NEC-associated regulator with potential therapeutic implications.

## Introduction

Necrotizing enterocolitis (NEC) is a severe gastrointestinal disease among preterm infants. Despite significant advances in neonatal medicine, NEC remains a major clinical challenge due to its high morbidity and mortality (1–3). The pathogenesis of NEC is multifactorial and complex. Therefore, elucidating the molecular mechanisms underlying NEC and identifying disease-associated regulatory factors are critical for improving our understanding of disease progression and developing potential therapeutic strategies. Disruption of the immune microenvironment and the impairment of intestinal barrier function are considered as key factors in the pathogenesis of NEC (4–7). In the immature intestine of preterm neonates, defective barrier function and dysregulated host-microbe interactions render the mucosa particularly susceptible to inflammatory injury (8). Excessive activation of innate and adaptive immune responses can further amplify tissue damage, disrupt tight junction integrity, and promote epithelial cell death (9). Multiple immune cells, including neutrophils, macrophages, T cells and dendritic cells (DCs), have been implicated in the immune dysregulation associated with NEC (7, 10–13). However, the precise mechanisms by which different cellular populations interact to shape the intestinal immune microenvironment remain incompletely understood.

In NEC, emerging evidence suggests that immune cells play critical roles in coordinating mucosal inflammation and epithelial injury (4, 10, 14). However, the key cellular mediators and signaling interactions driving these pathogenic processes have not been fully defined. In particular, the contribution of DCs, which serve as professional antigen-presenting cells and central regulators of intestinal immune homeostasis, remains unclear in NEC. Identification of the key genes and signaling pathways implicated in NEC pathogenesis is critical for improving mechanistic understanding. Notably, vitamin D3 has been reported to ameliorate intestinal injury by modulating DC maturation and activation through regulation of CD80/CD86 and indoleamine 2,3-dioxygenase 1 (IDO1) expression. In patients with NEC, activated DCs and neutrophils accumulate in the mucosa, indicating their migration from the periphery to inflamed area (15). This migration is linked to the upregulation of SELE, a gene that plays critical role in leukocyte recruitment, and elevated intestinal SELE levels has been linked to poor prognosis in NEC. In neonatal mice, C. sakazakii-induced DC recruitment in the lamina propria disrupts tight junctions and increases epithelial apoptosis, with DC depletion protecting against NEC and adoptive transfer of DCs enhancing infection susceptibility (16–18). Recent evidence also suggests that targeting innate inflammatory pathways, including MD2/TLR4 signaling, may attenuate intestinal inflammation and NEC severity (19). Collectively, these findings support a potential role for DC-centered immune dysregulation in NEC pathogenesis and underscore the need for further mechanistic investigation. In parallel, integrative bioinformatics approaches combining single-cell RNA sequencing (scRNA-seq), RNA sequencing (RNA-seq), and machine learning algorithms have emerged as powerful tools for identifying robust biomarkers and pathogenic regulators in human disease.

In the present study, we aimed to characterize the single-cell transcriptomic landscape of NEC, with a particular focus on DCs and their interactions with other immune and epithelial cell populations. We further applied machine learning approaches to identify candidate NEC-associated genes and explored immune infiltration features associated with disease progression. Our findings suggest that DCs may serve as important mediators of NEC-associated immune communication, while ALPK2 may represent a promising candidate regulator of immune microenvironment alterations and epithelial barrier dysfunction. These findings provide new insights into the molecular mechanisms underlying NEC and identify potential avenues for therapeutic exploration.

## Methods

### Data sources and processing

Transcriptomic data for NEC were obtained from the NCBI Gene Expression Omnibus (GEO) database GSE64801, including 5 controls and 9 NEC samples. scRNA-seq data were retrieved from the Zenodo repository (https://doi.org/10.5281/zenodo.5813397), including 11 samples (5 controls and 6 NEC samples). Single-cell expression matrices were imported into the Seurat package for quality control and downstream analysis. Core cells were filtered according to the total number of unique molecular identifiers (UMIs), the number of detected genes, and the percentage of mitochondrial transcripts. Quality control was performed using the median absolute deviation (MAD), and values located more than 3 MADs were defined as outliers and removed. Doublets were further identified and excluded for each sample using DoubletFinder (v2.0.4). The Gene Set Cancer Analysis (GSCA) Database was employed to predict drug targets associated with the identified marker genes. Afterwards we applied global-scaling normalization method “LogNormalize” for data normalization. The top 2,000 highly variable genes (HVGs) were selected using “FindVariableFeatures” function. Principal component analysis (PCA) was performed for linear dimension reduction using “RunPCA” function based on 2000 HVGs. Batch correction and integration of the samples were carried out using the “harmony” package. Dimensionality reduction and visualization was performed using uniform manifold approximation and projection (UMAP) with the “RunUMAP” functions. To construct the marker gene set, we use two comprehensive public single-cell databases-CellMarker database and PanglaoDB database. Finally, cell annotation was determined by the R package “singleR”.

### Cell–cell communication analysis

Intercellular communication analysis was performed using the CellChat package for scRNA-seq. Standardized single-cell gene expression profiles were used as input data, and cell subtypes identified by single-cell analysis were used as cellular annotations. The strength and extent of cell–cell interactions were quantified on the basis of interaction weights and interaction counts.

### Machine learning

LASSO regression and random forest (RF) machine learning algorithms were performed using the 14 samples (GSE64801; Control=5; NEC = 9) included in this study. Differentially expressed genes in DCs were identified using the FindAllMarkers function. Genes with adjusted p-value < 0.05 and average log2 fold change > 0.585 were considered significantly differentially expressed. The identified DC-associated differentially expressed genes were subsequently used as input features for exploratory feature selection using LASSO regression and RF algorithms. LASSO regression was performed using the “glmnet” package in R. LASSO regression with L1 regularization was used for feature selection, and genes with non-zero coefficients were retained as predictive features. LASSO regression was optimized using 10-fold cross-validation (nfold=10). All lambda values were evaluated, and the optimal penalty parameter (lambda.min) was selected based on the minimum classification error. Only genes with non-zero coefficients under the optimal lambda value were retained as predictive features, thereby reducing the risk of overfitting. RF analysis was subsequently performed as an independent feature-ranking approach. The percentage of increased mean-squared error (%IncMSE) was used as measurement of feature importance and the top 10 genes were selected. A total of 1000 decision trees (ntree=1000) were constructed to reduce random error across, and permutation-based feature importance analysis (nPerm=500) was performed to evaluate feature importance. Finally, the intersection of genes with non-zero coefficients from LASSO and those with high importance rankings from RF was taken as the key genes for further analysis.

### Immune infiltration analysis and functional annotation

The immune infiltration landscape in NEC and control samples from the GSE64801 dataset was evaluated using the CIBERSORT algorithm. CIBERSORT determined the proportions of 22 different infiltrating immune cell types. Correlation analysis was further performed to examine the relationships between hub gene expression and immune cell infiltration. Cytokine-related genes, including immunoinhibitors, immunostimulators, chemokines, and receptors, were included to examine the Spearman correlation with hub genes. Subsequently, we obtained related genes from the Molecular Signatures Database v7.0 (MSigDB). And gene set enrichment analysis (GSEA) was used to clarify the enriched KEGG pathways. Adjusted P-value < 0.05 was considered statistically different. We also performed a gene set variation analysis using the single gene-gene set variation analysis (GSVA). Additionally, AUCell was performed to assess enrichment scores for specific gene sets in scRNA-seq data.

### Regulatory network analysis of key genes

Disease data were retrieved from OMIM database (https://omim.org/). To further investigate disease-associated regulatory relationships, co-expression network analysis was performed with the hub genes and disease-related genes. Correlation analysis between the expression levels of hub genes and disease-related genes, where P<0.05 was considered statistically significant.

### Establishment of NEC model

SPF C57BL/6J mice, aged 5 days were obtained from Hangzhou Qizhen Laboratory Animal Co., Ltd. All mice were maintained in a pathogen-free environment with a regulated light/dark cycle.

The animal experiments were performed according to Guide for the Care and Use of Laboratory Animals, published by the US National Institute of Health and supervised by the Animal Care Committee of the Children’s Hospital of Shanghai. Experimental NEC model was performed as previously described. The modeling procedure was continued for 96 hours. At the experimental endpoint, all mice were euthanized by cervical dislocation in accordance with the approved institutional animal care protocol. The intestinal tissues were immediately harvested for subsequent analyses.

### Intestinal organoid culture and treatment

The establishment of the mouse intestinal organoid model was performed as previously reported in (20), with minor modifications. The resulting organoids were treated with complete medium (#K2501-MIW, bioGenous). An *in vitro* inflammation model was induced in intestinal organoids. organoids were exposed to lipopolysaccharide (LPS) with 100 µg/mL (#L4524, Sigma-Aldrich) for 72h. After LPS stimulation, 20 µM TGX221 (#HY-10114, MedChemExpress) were added in TGX221-treated groups 72h. Organoids were collected for subsequent analysis.

### Histology and immunofluorescence staining

Organoids or intestinal tissue sections were fixed with 4% paraformaldehyde for overnight. Then, embedded in paraffin and sectioned. Next, hematoxylin and eosin (H&E) staining was performed to assess intestinal morphology. The staining process included hematoxylin staining to visualize cellular nuclei and eosin staining to highlight cytoplasmic structures. For immunofluorescence experiments, antigen retrieval of sections was performed by Tris-EDTA buffer. Next, organoids were permeabilized with 0.3% Triton X-100 for 1 h at room temperature. The intestinal organoids were then incubated overnight at 4°C with primary antibodies against ZO-1 (#21773-1-AP, PTG) and claudin-1 (#ab307692, abcam). After washing three times in PBS, incubated with Goat Anti-Rabbit IgG H&L (HRP) antibodies (#ab205718, abcam) for 45minutes, DAPI stained (Vector Laboratories Inc) for 5 minutes, and mounted. The images were captured using a microscope.

### Quantitative real-time PCR analysis

Total RNA was extracted from intestinal tissues or organoid samples using TRIzol reagent (#15596026, Invitrogen) according to the manufacturer’s instructions. RNA purity weas quantified using a NanoDrop spectrophotometer. cDNAs were synthesized using an EvoM-MLV RT Premix kit (#AG11706, AG). qRT-PCR was performed using a Hieff UNICON qPCR SYBR Green Master Mix kit (#11198ES08, Yeasen). β-actin was used as an internal control. The primers used are shown in **Supplementary Table 1**.

### Statistical analysis

All statistical analyses were conducted using the R programming language(version 4.3.0). All correlation tests were conducted using the pearson and spearson correlation analysis method in the study. For comparisons between two groups with a single variable, Mann-Whitney test was applied. Differences among more than two groups were evaluated using one-way ANOVA with Tukey’s *post-hoc* test or Kruskal-Wallis test. *p<0.05, **p<0.01, ***p<0.001 were set as statistically significant.

## Results

### scRNA-seq analysis identifies DC-centered communication networks in NEC

**Figure 1:**
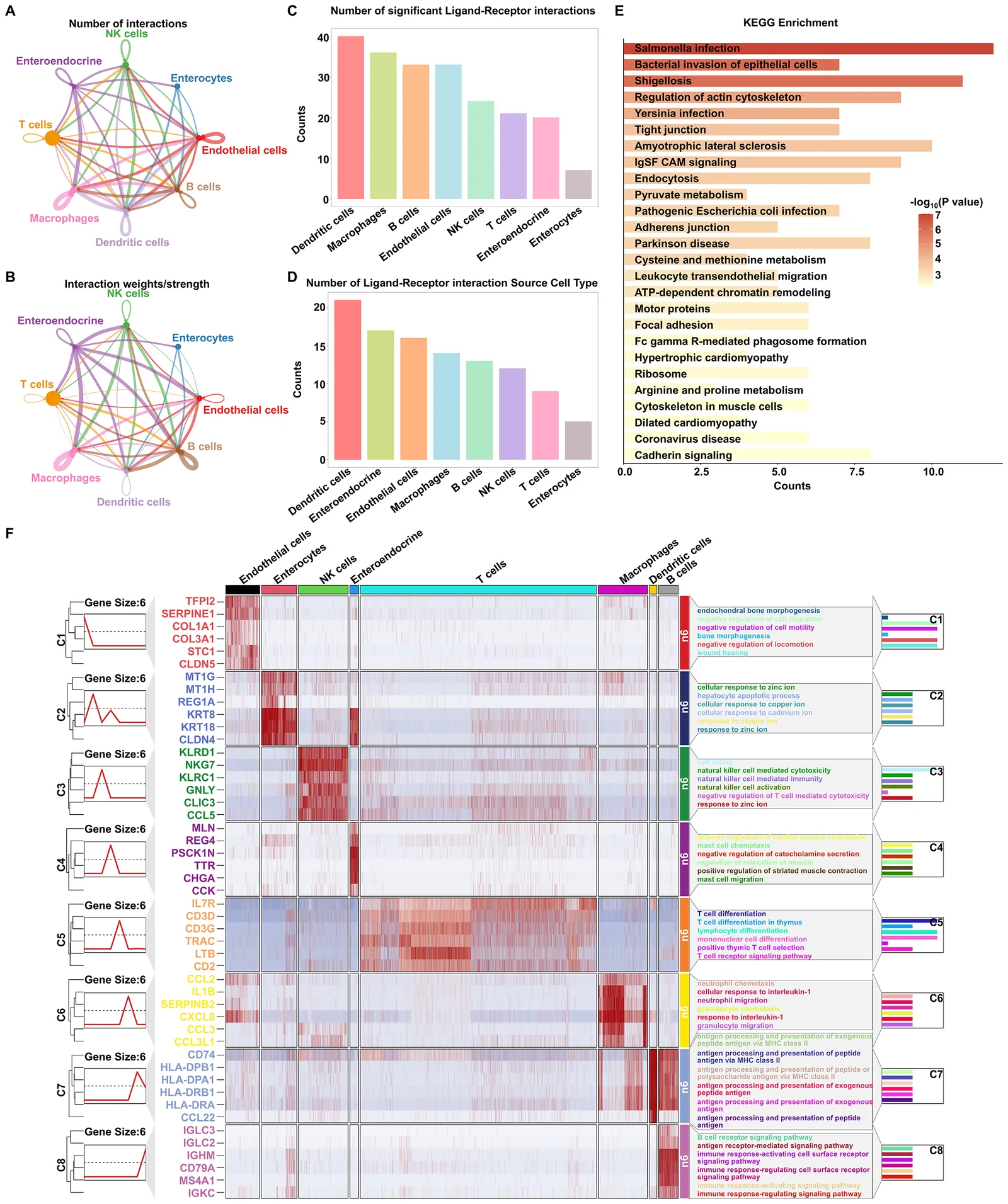
DCs occupy a central role in NEC intercellular communication. **(A)** Circle plot showing the number of inferred intercellular interactions among the 8 major cell populations. **(B)** Circle plot showing the interaction weights/strengths among the 8 major cell populations. **(C)** Bar plot showing the number of significant ligand–receptor interactions identified among different cell populations. **(D)** Bar plot showing the number of outgoing ligand–receptor interactions from each cell cluster. **(E)** KEGG pathway enrichment analysis of differentially expressed genes between DCs from control and NEC samples. **(F)** Heatmap showing representative cluster-specific marker genes across the annotated cell populations, together with functional enrichment analysis of the corresponding gene sets.

To visualize the overall distribution of single cells, we performed t-SNE dimensionality reduction on the control and NEC groups. Following quality control, 12,039 cells were retained for subsequent analysis (**Supplementary Figures 1A, B**). The top 2000 HVGs were identified and performed standardization, homogenization, PCA, and Harmony analysis on the data in sequence (**Supplementary Figures 1C–F**). Eight major cell populations were identified, including Endothelial cells, Enterocytes, NK cells, Enteroendocrine cells, T cells, Macrophages, DCs, B cells (**Supplementary Figures 2A–C**). Immune cells represented the predominant population within the NEC single-cell atlas (**Supplementary Figure 2D**). To investigate disease-associated intercellular communication patterns, we performed CellChat analysis across the annotated cell populations in control and NEC group. Extensive communication networks were observed among different cell subtypes (**Figures 1A, B**). DCs showed the highest number of inferred interaction pathways among the annotated cell types. (**Figures 1C, D**). Given that DCs play a critical regulatory role in NEC-associated cellular communication, we further characterized DC-specific transcriptional alterations. KEGG analysis of differentially expressed genes between DCs from control and NEC samples enriched pathways in intestinal barrier regulation, pathogen recognition, and leukocyte inflammatory migration (**Figure 1E**). Furthermore, the top six marker genes in the DCs cluster were predominantly associated with antigen processing and presentation pathways, including MHC class II-mediated antigen presentation, highlighting the involvement of DCs in antigen presentation and immune activation within the NEC-associated immune microenvironment (**Figure 1F**).

### Identification of the key genes by machine learning

**Figure 2:**
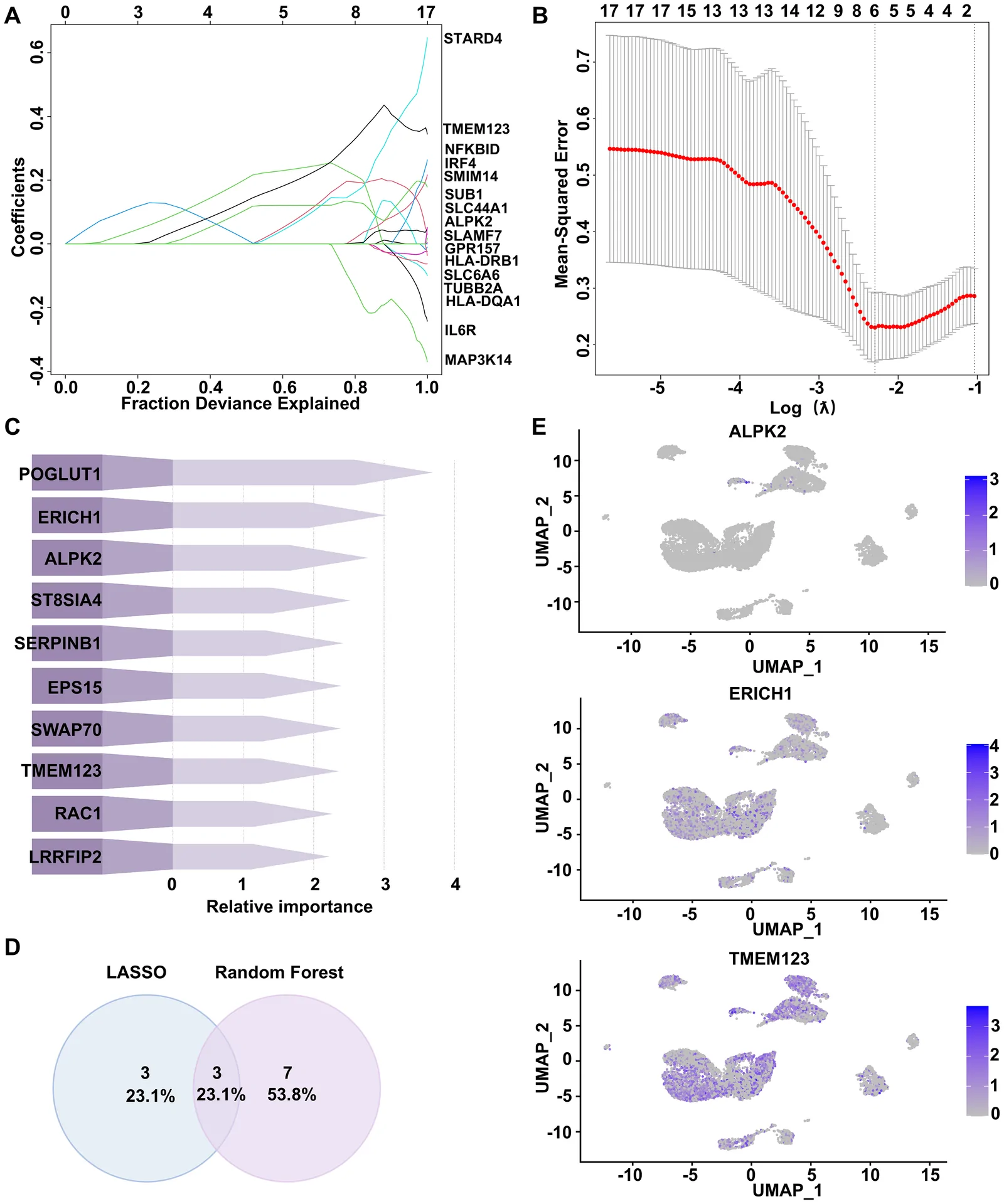
Machine learning-based screening identifies NEC-associated hub genes in DCs. **(A)** LASSO coefficient profiles of candidate genes associated with NEC. The different colors of the lines represent different genes. **(B)** LASSO regression analysis examining the relationship between deviance and the logarithm of lambda. **(C)** RF analysis showing the top 10 genes ranked by relative importance. **(D)** Venn diagram showing the overlap genes selected by LASSO regression and RF analysis. **(E)** Expression of hub genes (ALPK2, ERICH1, and TMEM123) for each cluster.

To further identify candidate NEC-associated genes, we analyzed the differentially expressed genes in DCs between NEC and control samples. A total of 427 differentially expressed genes in DCs were identified and subsequently subjected to feature selection by LASSO regression and RF (**Supplementary Table 2**). Through LASSO regression, six genes were pinpointed as characteristic of NEC (**Figures 2A, B**). The top 10 genes identified by the RF algorithm are shown in **Figure 2C**, namely POGLUT1, ERICH1, ALPK2, ST8SIA4, SERPINB1, EPS15, SWAP70, TMEM123, RAC1 and LRRFIP2. After the intersection, three hub genes shared by the LASSO regression algorithm and the RF algorithm were finally confirmed ERICH1, ALPK2 and TMEM123 (**Figure 2D**). Subsequently, the overlapping genes were selected as candidate NEC-associated genes for further analyses. We also assessed the expression levels of three core immune-related genes in single cells. TMEM123 showed particularly pronounced expression (**Figure 2E**).

### Analysis of immune microenvironment characteristics and key genes

**Figure 3:**
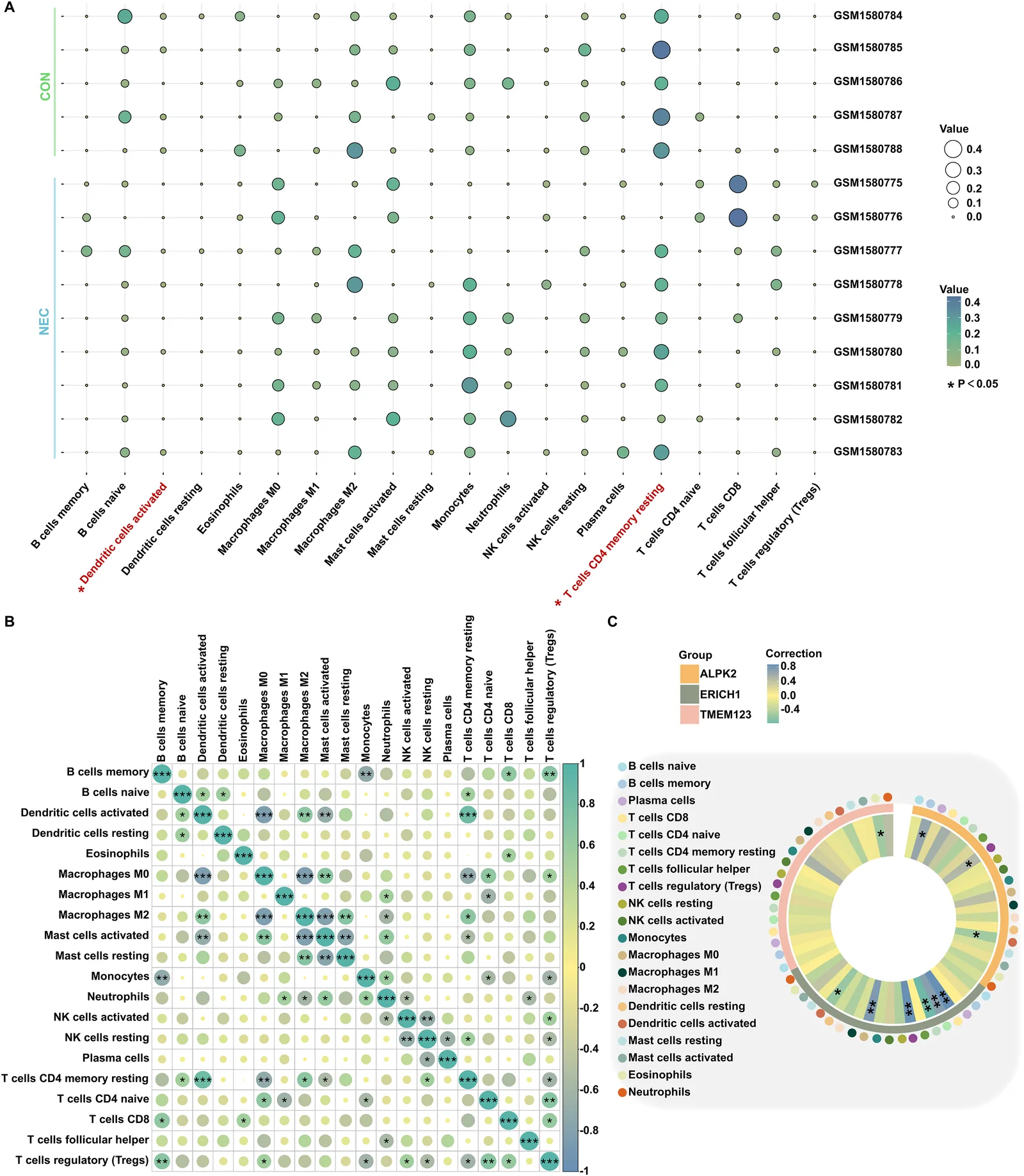
Immune microenvironment characteristics altered in NEC. **(A)** Distribution of different type of immune cells between control and NEC group. Each column represents an immune cell type, and each row represents an individual sample. The size and color intensity of the bubbles represent the relative proportion of immune cell subsets. Statistical significance was defined as P < 0.05. **(B)** Correlation of each type of immune cells. **(C)** Correlation heatmap showing the associations between the identified hub genes (ALPK2, ERICH1, and TMEM123) and 22 types of immune cells. *P < 0.05, **P < 0.01, ***P<0.001.

The immune microenvironment plays a critical role in the pathogenesis and progression of NEC. Therefore, we applied the CIBERSORT algorithm to evaluate the infiltration patterns of 22 immune cell types using the NEC dataset (GSE64801). As shown in **Figure 3A**, there is a significant difference in the immune cell profiles between the NEC group and the control group. Compared to controls, NEC tissues showed lower levels of DCs activated and T cells CD4 memory resting. **Figure 3B** displays heatmaps of correlations between different immune cells, further indicating that the NEC group exhibits a distinct immune profile compared to the control group, as well as highlighting the interactions among various types of immune cells (**Supplementary Table 3**). Additionally, we explored the associations between core immune-related genes and immune cell components. The results demonstrated that ALPK2 was significantly positively correlated with B cells memory and T cells CD8, while showing a significant negative correlation with DCs activated. ERICH1 exhibited significant positive correlations with T cells CD8, T cells CD4 naive, regulatory T cells and Macrophages M0, but was significantly negatively correlated with T cells CD4 memory resting and DCs activated. In contrast, TMEM123 showed a significant negative correlation with eosinophil infiltration (**Figure 3C**; **Supplementary Table 4**). Furthermore, we performed a comprehensive correlation analysis to explore the associations between the identified key genes and a broad spectrum of immune-related factors, including immunosuppressive factors, immunostimulatory factors, chemokines, and receptors (**Supplementary Figures 3A–E**).

### Pathway enrichment analysis of key DCs genes

**Figure 4:**
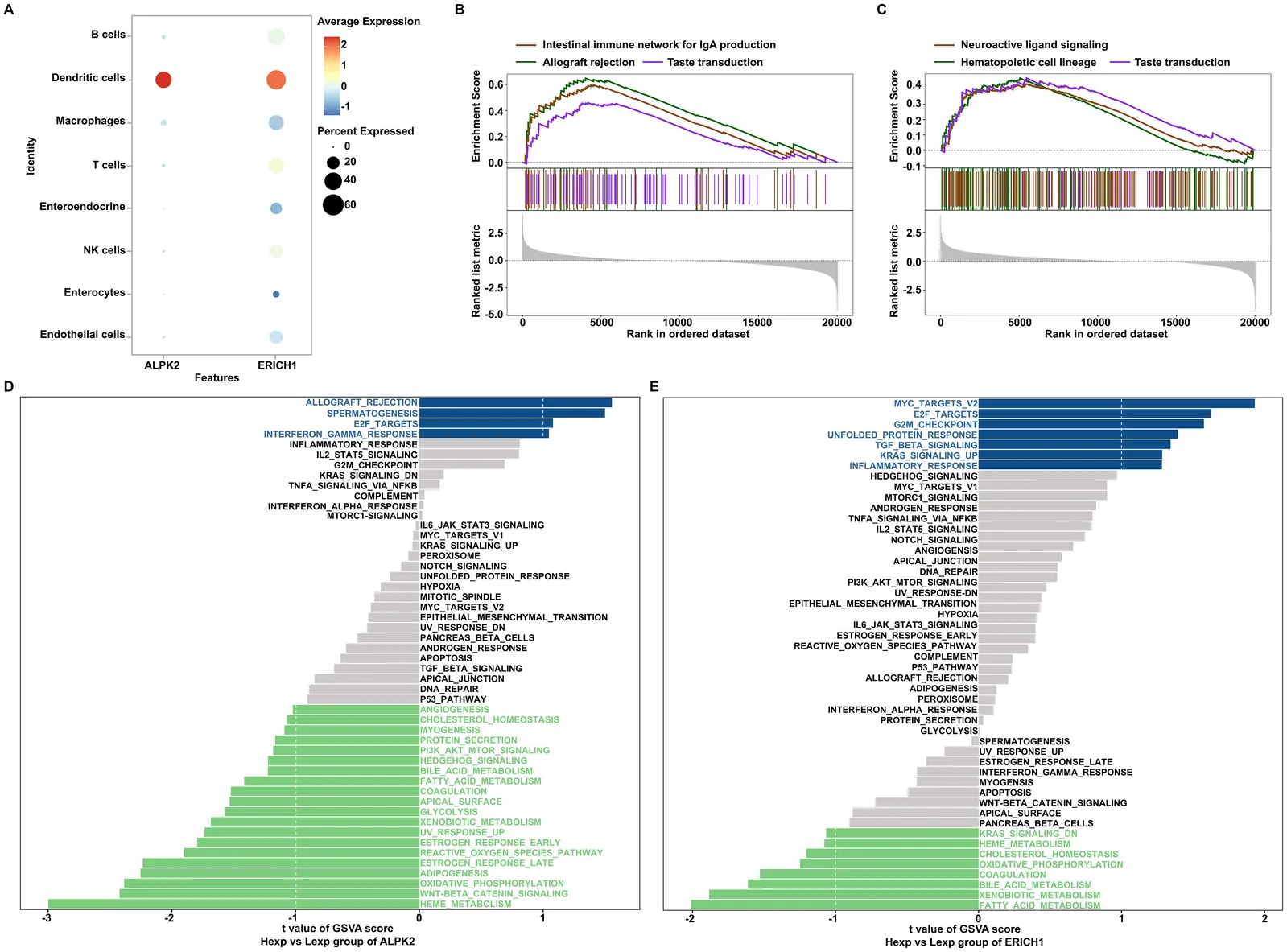
Pathway enrichment analysis of key DCs-associated genes. **(A)** Dot plot showing the expression patterns of ALPK2 and ERICH1 in the single-cell atlas. **(B)** GSEA of ALPK2-associated signaling pathways. **(C)** GSEA of ERICH1-associated signaling pathways. **(D)** GSVA of significantly enriched pathways in high- and low-expression groups of ALPK2 based on the Hallmark gene set. **(E)** GSVA of significantly enriched pathways in high- and low-expression groups of ERICH1 based on the Hallmark gene set.

We conducted single-cell validation to identify the cell types affected by these two genes. This analysis confirmed that ALPK2 and ERICH1 were highly expressed in DCs (**Figure 4A**). Next, we analyzed specific signaling pathways involving key genes closely associated with DCs and explored the effect of candidate genes on the signaling pathways related to disease progression. GSEA results showed that ALPK2 enriched in allograft rejection, intestinal immune network for IgA production, taste transduction and so on (**Figure 4B**). ERICH1 enriched in neuroactive ligand signaling, taste transduction and hematopoietic cell lineage (**Figure 4C**). In addition, we also performed GSVA analysis on these genes. The results showed that high expression of ALPK2 mainly enriched in allograft_rejection, spermatogenesis and other signal pathways (**Figure 4D**). The enrichment of these signaling pathways suggests that ALPK2 may not only influence immune rejection responses but also participate in disease-associated immune reactions through the regulation of reproductive processes. In parallel, high expression of ERICH1 mainly enriched signaling pathways such as myc_targets-v2, e2f_targets and so on (**Figure 4E**). ERICH1 may exert functional effects within the immune microenvironment by modulating cell-cycle related pathways and transcriptional regulatory networks governing gene expression.

### Disease-relevant co-expression network of key regulatory genes

**Figure 5:**
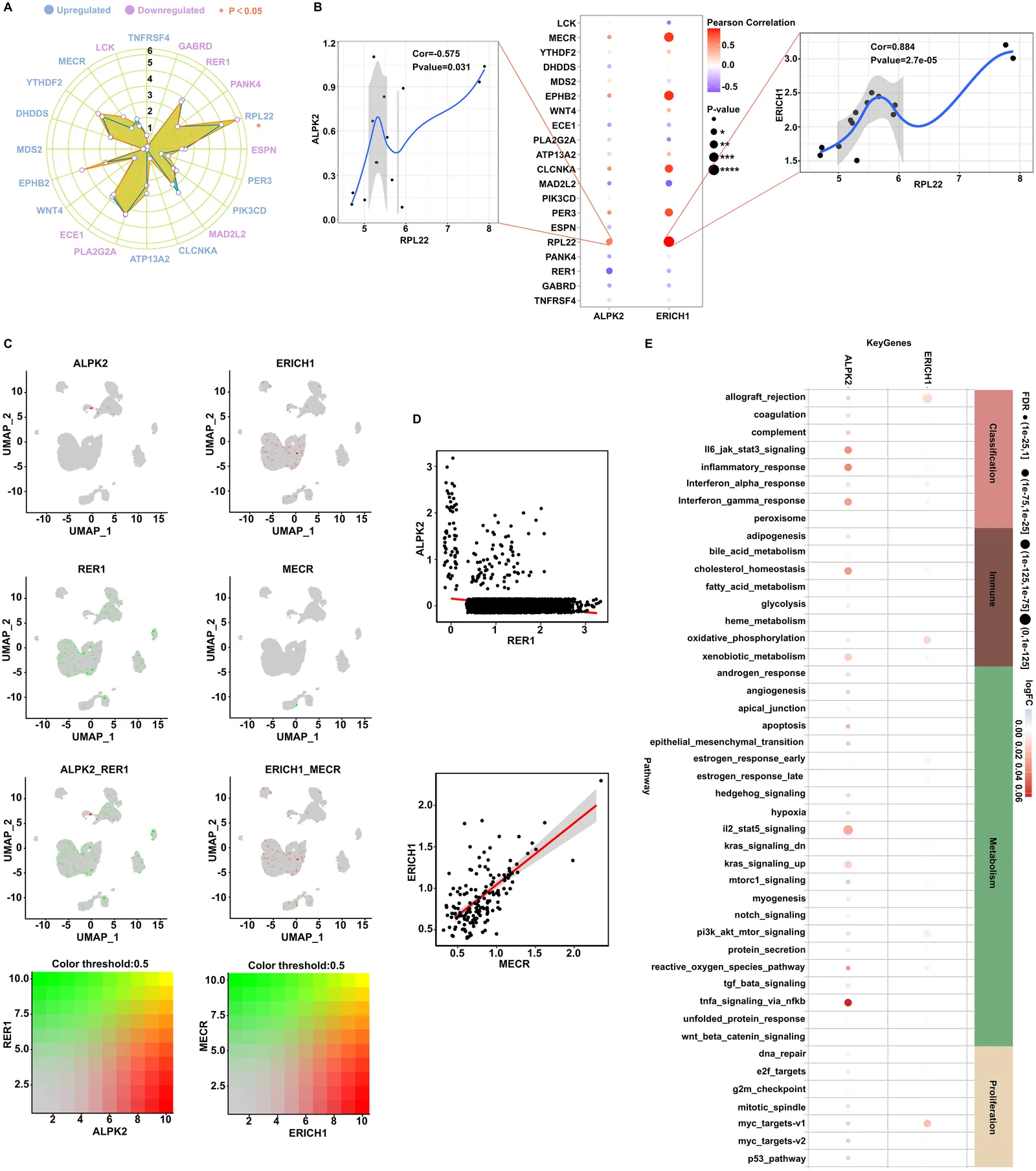
Disease-associated co-expression patterns and signature activity of key genes in NEC. **(A)** Comparison of disease-related genes in control and NEC groups. **(B)** Pearson correlation analysis of hub genes (ALPK2, ERICH1) and disease-associated gene RPL22. **(C)** The co-expression of ALPK2-RER1 and ERICH1-MECR on the UMAP. **(D)** Scatter plots showing the correlations of ALPK2 with RER1 and ERICH1 with MECR. **(E)** AUCell analysis showing the activity of ALPK2 and ERICH1 related Hallmark gene signatures.

Selected the top 20 genes most relevant to NEC from the OMIM database. The results showed the expression of RPL22 was significant different between control group and NEC group (**Figure 5A**). The pearson correlation analysis revealed that the hub genes ALPK2 and ERICH1 were significantly associated with the expression levels of the gene RPL22. Specifically, ERICH1 showed a significant positively correlation with RPL22 (r=0.884), whereas ALPK2 exhibited a significant negatively correlation with RPL22 (r=−0.575) (**Figure 5B**). Correlation analyses were conducted to characterize the co-expression network between RER1 and MECR with the key genes at the single-cell level. (**Figures 5C, D**). Additionally, Using AUCell, we quantified the activity of immune and metabolism related gene signatures at the single-cell level based on Hallmark gene sets. ERICH1 displayed pronounced activity in metabolic-related pathways indicating a functional profile that is mechanistically distinct from the immunomodulatory role of ALPK2. In contrast, ALPK2 showed markedly higher activity in immune- and inflammation-related pathways, including TNF-α signaling via NF-κB and inflammatory response (**Figure 5E**). These findings support a central role for ALPK2 in immune regulation and inflammatory signaling, and may be critically involved in the pathogenesis of inflammation-driven diseases such as NEC.

### Role of ALPK2 in NEC-like injury of intestinal organoids

**Figure 6:**
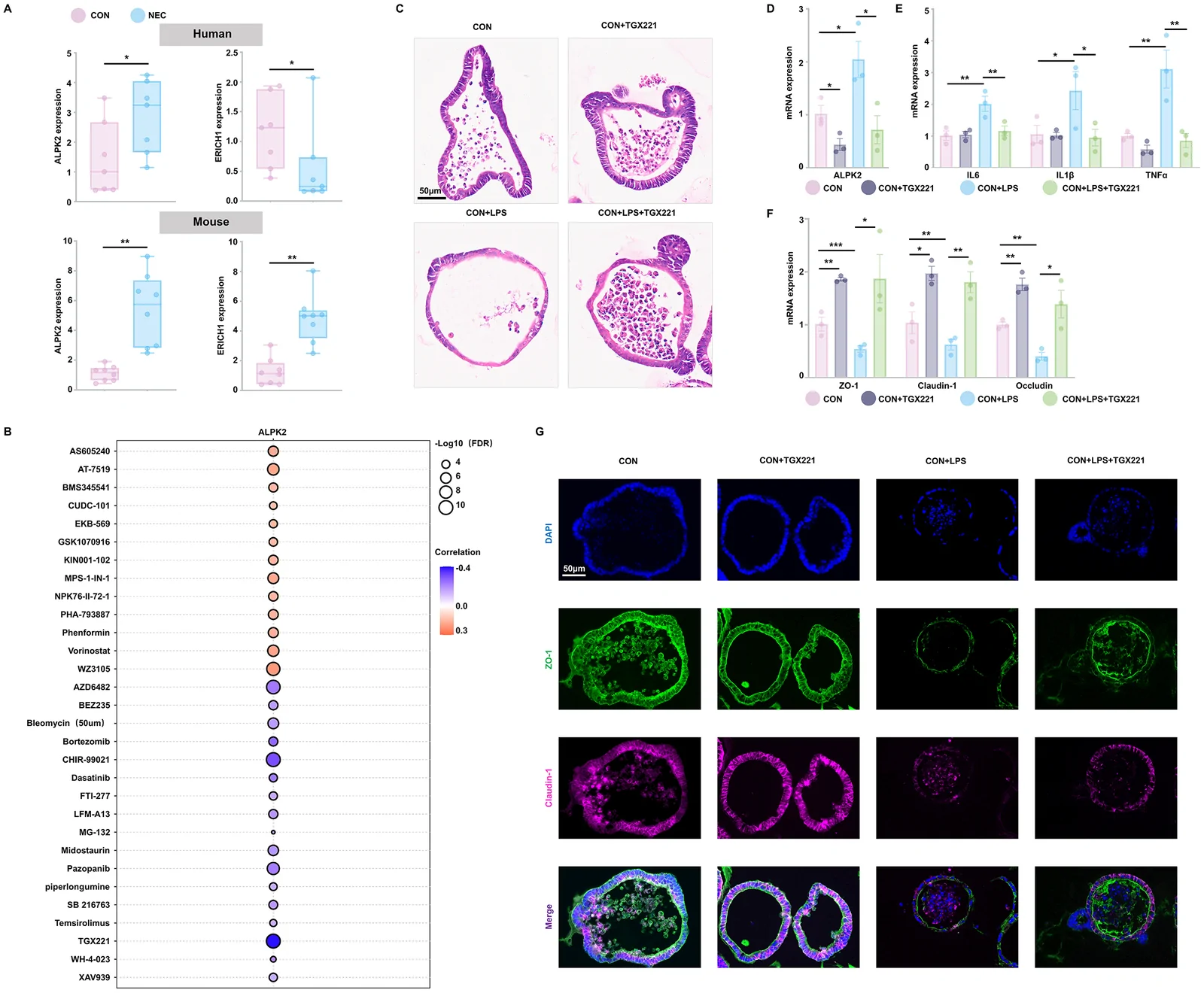
ALPK2 inhibition attenuates inflammatory injury and improves epithelial barrier integrity. **(A)** mRNA expression of ALPK2 and ERICH1 in ileal tissues from human and mice (human:control = 7, NEC = 7; mice: control = 8, NEC = 8). Data are presented as mean ± SEM. **(B)** GSCA-based drug sensitivity analysis for ALPK2. **(C)** Representative histological images of mouse intestinal organoids in different group. Scale bars, 50 μm. **(D)** qRT-PCR analysis of ALPK2 in mouse intestinal organoids under the indicated treatment conditions. Data are presented as mean ± SEM. **(E)** qRT-PCR analysis of inflammatory cytokines (IL-6, IL-1β, and TNF-α) in mouse intestinal organoids under the indicated treatment conditions. Data are presented as mean ± SEM. **(F)** qRT-PCR analysis tight junction-related genes (ZO-1, claudin-1, and occludin) in mouse intestinal organoids under the indicated treatment conditions. Data are presented as mean ± SEM. **(G)** Immunofluorescence staining of organoids showing DAPI (blue), ZO-1 (green), and claudin-1 (pink) under the indicated treatment conditions. Scale bars, 50 μm. *P < 0.05, **P < 0.01, ***P<0.001.

Although ERICH exhibited partial co-expression patterns and showed activity within certain immune metabolic pathways, integrative analyses indicated that ALPK2 is more directly involved in NEC pathogenesis, primarily through immune and inflammatory signaling cascades. qRT-PCR analysis revealed that ALPK2 expression was significantly elevated in ileal tissues from both NEC patients and experimental NEC mouse models compared with controls (**Figure 6A**). Baseline demographic and clinical characteristics of infants with NEC and controls are presented in **Supplementary Table 5**. To assess the functional relevance of ALPK2, GSCA database analysis identified TGX221 as the most sensitive compound to ALPK2 (**Figure 6B**). We employed intestinal organoids to assess TGX221 therapeutic potential in repairing NEC-like injuries. We observed that TGX221 decreased ALPK2 and inflammatory cytokines expression in a time- and dose-dependent manner, with 20μM TGX221 treatment for 72h significantly suppressed their expression levels (**Supplementary Figures 4A–C**). TGX221 treatment attenuated LPS-induced intestinal organoid injury at the optimal concentration of 20μM for 72h (**Figure 6C**). Compared with the control group, LPS stimulation significantly increased mRNA expression ALPK2. Conversely, TGX221 supplementation remarkably reversed this tendency (**Figure 6D**). Notably, suppression of ALPK2 was accompanied by a significant decrease in IL-6, IL-1β and TNF-α level, which were ever lower with TGX221 treatment (**Figure 6E**). In parallel, TGX221 treatment significantly increased the mRNA expression of the tight junction proteins zo-1, occludin, and claudin-1, suggesting restoration of intestinal epithelial barrier integrity (**Figure 6F**). Consistently, IF staining demonstrated that TGX221 preserved tight junction organization disrupted by LPS exposure (**Figure 6G**). Collectively, these findings identify ALPK2 as a pivotal regulator of intestinal inflammation and mucosal damage repair.

## Discussion

NEC remains one of the most life-threatening gastrointestinal disorders in premature infants and contributes substantially to neonatal morbidity and mortality (2, 3, 7). Despite extensive research, its pathogenesis is still unclear. Therefore, identifying disease-associated molecular signatures and key regulatory factors is essential for improving our understanding of NEC pathogenesis and exploring potential therapeutic strategies. In this study, we integrated scRNA-seq and RNA-seq datasets to elucidate NEC-associated immune dysregulation, highlighting DCs-centered intercellular communication networks and immune-related molecular signatures. Immune-related genes identified through integrative bioinformatic analyses may represent candidate NEC-associated regulators, warranting further validation in larger cohorts and functional studies.

Increasing evidence indicates that NEC pathogenesis is not driven by epithelial injury alone, but rather by the convergence of mucosal immune dysregulation, inflammatory amplification, and intestinal barrier failure (4, 5, 7, 21). Our analysis revealed alterations in the immune landscape of NEC and identified DCs as an important component of the NEC-associated intercellular communication network. As specialized antigen-presenting cells, DCs play essential roles in coordinating adaptive immune responses and maintaining intestinal immune homeostasis (18, 22–24). Consistent with their central role in immune communication, NEC-associated DCs exhibited distinct transcriptional alterations relative to control DCs. KEGG analysis revealed that these differentially expressed genes were primarily enriched in pathways associated with intestinal barrier regulation, pathogen recognition, and leukocyte inflammatory migration, suggesting functional remodeling of DCs in response to the inflammatory microenvironment of NEC. NEC disrupts intestinal immune homeostasis through exaggerated innate cytokine responses, dysregulated adaptive immune responses, and impaired regulatory mechanisms, ultimately contributing to epithelial barrier dysfunction and inflammatory amplification (25). Previous studies have shown that pathogen-induced DC recruitment and impaired DC maturation may contribute to epithelial barrier dysfunction in NEC by modulating the expression and integrity of tight junction proteins (16). Collectively, our findings further support an important role for DCs in NEC-associated immune communication network and suggest that NEC-associated transcriptional alterations in DCs may be associated with inflammatory responses and intestinal barrier dysfunction.

Through integrative machine learning analyses, we identified ERICH1, ALPK2, and TMEM123 as candidate NEC-associated hub genes. CIBERSORT analysis revealed significant alterations in immune cell composition in NEC samples, including a marked reduction in activated DCs. Interestingly, ALPK2 and ERICH1 expression was negatively correlated with activated DCs, suggesting that dysregulation of these hub genes may be associated with changes in DC-related immune responses and the inflammatory balance in NEC. Single-cell analysis further confirmed that ALPK2 and ERICH1 are predominantly expressed in DCs, implicating these genes in DC-mediated immune regulation within the intestinal microenvironment. Pathway enrichment analyses revealed distinct functional associations of the identified candidate genes. ALPK2 was primarily associated with immune-related pathways, including mucosal immune networks and allograft rejection pathways. These findings suggest that ALPK2 may be involved in DC-associated immune activation and inflammatory amplification during NEC progression. In contrast, ERICH1 was mainly associated with transcriptional and proliferative pathways, including MYC and E2F target programs. Together, these findings indicate that NEC involves alterations in immune signaling, metabolic processes, and transcriptional regulation, with ALPK2 representing a candidate NEC-associated regulator requiring further investigation.

To identify key NEC-related genes, we selected the top 20 candidates from the OMIM database, among which RPL22 was significantly dysregulated between NEC and control samples. While traditionally recognized as a ribosomal component, RPL22 has been increasingly implicated in immune regulation and cellular stress responses, including the regulation of αβ T cell and B cell lineage differentiation through p53-dependent checkpoints and endoplasmic reticulum stress pathways (26, 27). In our study, ALPK2 expression was negatively correlated with RPL22, whereas ERICH1 showed a positive correlation with RPL22, suggesting that these genes may reflect distinct molecular alterations associated with NEC-associated immune dysregulation. Together, these findings indicate that NEC pathogenesis involves alterations across multiple cellular and molecular processes Mechanistically, single-cell AUCell analysis revealed distinct functional associations of ALPK2 and ERICH1. ERICH1 showed higher activity in metabolic-related pathways, suggesting its potential association with metabolic processes and transcriptional regulation. In contrast, ALPK2 enriched in immune and inflammatory pathways, including TNF-α signaling via NF-κB and inflammatory response signatures, supporting its potential involvement in NEC-associated immune activation and inflammatory responses. Although direct evidence of ALPK2 in intestinal immunity are limited, studies of alpha kinase family members suggest that this protein family may participate in gut inflammatory regulation. For instance, ALPK1 has been reported to modulate colitis severity through regulation of IL-12/Th1 responses, while ALPK2 has been implicated in epithelial homeostasis, including apoptosis and DNA repair (28–30). In summary, these findings suggest that dysregulation of ALPK2 and ERICH1 may be involved in NEC-associated immune microenvironment alterations and epithelial barrier dysfunction, highlighting their potential relevance for future mechanistic studies and therapeutic exploration in NEC.

Consistent upregulation of ALPK2 was observed in intestinal tissues from both NEC patients and mice, indicating its crucial role in the development of NEC. Therefore, ALPK2 was selected for further analysis. Notably, premature infants have immature intestinal defense mechanisms, rendering the gut particularly susceptible to microbial stimulation and barrier disruption (31, 32). Previous studies have demonstrated that DC recruitment and activation may promote local cytokine production, including TGF-β, potentially leading to epithelial apoptosis and impaired intestinal barrier integrity, thereby contributing to NEC-associated epithelial injury (16). Using GSCA as a pharmacogenomic screening resource, we identified TGX221 as the compound with the strongest predicted sensitivity relationship to ALPK2. In LPS-induced intestinal organoids, TGX221 treatment reduced ALPK2 expression, attenuated inflammatory cytokine induction, and partially restored tight junction protein expression. The results show that ALPK2 as a potential therapeutic target for modulating intestinal inflammation and promoting mucosal repair in NEC. Collectively, our findings suggest that ALPK2 may represent a candidate NEC-associated regulator involved in immune dysregulation and epithelial barrier dysfunction, providing a basis for further investigation of its biological function and therapeutic potential in NEC.

Although this study provides insights into NEC-associated immune dysregulation and identifies ALPK2 as a candidate NEC-associated regulator, several limitations should be acknowledged. First, some low-abundance cell populations may be underrepresented after stringent QC filtering. In particular, fibroblast-like cells did not form a distinct cluster in our analysis, which may be attributed to the exclusion of cells with fibroblast-associated transcriptional features during QC. Future studies using larger cohorts and optimized single-cell approaches are needed to further characterize fibroblast and other stromal cell populations in NEC. Second, the relatively small transcriptomic cohort used for machine-learning analysis (GSE64801; Control=5; NEC = 9) may limit the robustness of computational feature selection. Therefore, the identified genes should be considered candidate NEC-associated regulators requiring further validation rather than definitive diagnostic biomarkers. Larger cohorts and independent validation datasets are required to further evaluate their clinical relevance. Third, the human intestinal tissue cohort was limited by the availability of NEC surgical specimens. Although samples were carefully matched for key clinical characteristics, larger multicenter cohorts are required to further validate the clinical relevance of ALPK2 in NEC. Finally, although functional experiments demonstrated the association of ALPK2 with inflammatory responses and intestinal barrier disruption, its upstream regulatory mechanisms and precise role in DC-associated immune alterations remain to be further elucidated. In addition, the therapeutic relevance of TGX221 requires further validation through dedicated pharmacological studies and preclinical models.

Collectively, our study provides a coherent framework linking DC-centered communication, hub gene dysregulation, and epithelial barrier injury in NEC. Future studies should further define the regulatory mechanisms of ALPK2 in DCs and epithelial compartments and assess the therapeutic potential of targeting its associated pathways. These findings advance our understanding of NEC-associated immune dysregulation and provide a rationale for future investigations into mucosal immune modulation and epithelial barrier repair.

## Data Availability

The datasets presented in this study can be found in online repositories. The names of the repository/repositories and accession number(s) can be found in the article/**Supplementary Material**.

## Supplementary material

Supplementary Figure 1 Quality control and filtering of single cell data used for NEC analysis. **(A)** Single-cell quality control showing the UMI counts, gene count, mitochondrial content and ribosomal content for each sample. **(B)** The graph shows the relationship between UMI counts and gene number, mitochondrial content and ribosomal genes. **(C)** Characteristic variance plots for genes that differ significantly between cells. **(D)** Ranking plot of variance for each PC. **(E)** PCA used for downstream dimensionality reduction and clustering. **(F)** Harmony-integrated embedding demonstrating effective batch correction across samples.

Supplementary Figure 2 Screening of single-cell data. **(A)** UMAP plot showing that all cells were classified into 13 clusters. **(B)** UMAP of eight different cell types of samples. **(C)** Dot plot showing the marker genes used for cell type identification. **(D)** Distribution of annotated cell populations across individual samples (left), between the control and NEC groups (middle), and the total cell numbers for each cell type (right).

Supplementary Figure 3 Pearson correlation analysis of hub genes with immune-related genes in NEC. **(A–E)** Correlation analyses showing the associations of the identified hub genes with multiple immune-related factors, including chemokines-related genes **(A)**, receptors-related genes **(B)**, MHC-related genes **(C)**, immunoinhibitors-related genes **(D)**, immunostimulators-related genes **(E)**.

Supplementary Figure 4 TGX221 reduced ALPK2 and inflammatory cytokine expression in a dose- and time-dependent manner. **(A–C)** Expression levels of ALPK2 and inflammatory cytokines in LPS-induced intestinal organoids treated with different concentrations of TGX221 (0, 2.5, 5, 10, 20, and 25μM) for 24h (A), 48h **(B)**, and 72h **(C)**. Data are presented as mean ± SEM. *P < 0.05, **P < 0.01, ***P<0.001.

Supplementary Table 1 Primers used for qRT-PCR.

Supplementary Table 2 Differentially expressed genes identified in DCs using the FindAllMarkers function.

Supplementary Table 3 Correlation analysis among different immune cell populations. The correlation coefficient and corresponding P values are provided.

Supplementary Table 4 Correlation analysis between hub gene expression and immune cells. Correlation coefficients and corresponding P values are indicated.

Supplementary Table 5 Baseline characteristics of the infants in the control and NEC group.
